# The ORF59 DNA polymerase processivity factor homologs of Old World primate RV2 rhadinoviruses are highly conserved nuclear antigens expressed in differentiated epithelium in infected macaques

**DOI:** 10.1186/1743-422X-6-205

**Published:** 2009-11-18

**Authors:** A Gregory Bruce, Angela M Bakke, Courtney A Gravett, Laura K DeMaster, Helle Bielefeldt-Ohmann, Kellie L Burnside, Timothy M Rose

**Affiliations:** 1 Center for Childhood Infection and Prematurity Research, Seattle Children's Research Institute, 1900 Ninth Ave, Seattle, WA 98101-1304, USA; 2 School of Veterinary Science, University of Queensland, Brisbane, Qld, Australia; 3 Department of Pediatrics, University of Washington, Seattle, WA 98195, USA

## Abstract

**Background:**

ORF59 DNA polymerase processivity factor of the human rhadinovirus, Kaposi's sarcoma-associated herpesvirus (KSHV), is required for efficient copying of the genome during virus replication. KSHV ORF59 is antigenic in the infected host and is used as a marker for virus activation and replication.

**Results:**

We cloned, sequenced and expressed the genes encoding related ORF59 proteins from the RV1 rhadinovirus homologs of KSHV from chimpanzee (PtrRV1) and three species of macaques (RFHVMm, RFHVMn and RFHVMf), and have compared them with ORF59 proteins obtained from members of the more distantly-related RV2 rhadinovirus lineage infecting the same non-human primate species (PtrRV2, RRV, MneRV2, and MfaRV2, respectively). We found that ORF59 homologs of the RV1 and RV2 Old World primate rhadinoviruses are highly conserved with distinct phylogenetic clustering of the two rhadinovirus lineages. RV1 and RV2 ORF59 C-terminal domains exhibit a strong lineage-specific conservation. Rabbit antiserum was developed against a C-terminal polypeptide that is highly conserved between the macaque RV2 ORF59 sequences. This anti-serum showed strong reactivity towards ORF59 encoded by the macaque RV2 rhadinoviruses, RRV (rhesus) and MneRV2 (pig-tail), with no cross reaction to human or macaque RV1 ORF59 proteins. Using this antiserum and RT-qPCR, we determined that RRV ORF59 is expressed early after permissive infection of both rhesus primary fetal fibroblasts and African green monkey kidney epithelial cells (Vero) *in vitro*. RRV- and MneRV2-infected foci showed strong nuclear expression of ORF59 that correlated with production of infectious progeny virus. Immunohistochemical studies of an MneRV2-infected macaque revealed strong nuclear expression of ORF59 in infected cells within the differentiating layer of epidermis corroborating previous observations that differentiated epithelial cells are permissive for replication of KSHV-like rhadinoviruses.

**Conclusion:**

The ORF59 DNA polymerase processivity factor homologs of the Old World primate RV1 and RV2 rhadinovirus lineages are phylogenetically distinct yet demonstrate similar expression and localization characteristics that correlate with their use as lineage-specific markers for permissive infection and virus replication. These studies will aid in the characterization of virus activation from latency to the replicative state, an important step for understanding the biology and transmission of rhadinoviruses, such as KSHV.

## Introduction

Multiple proteins encoded in herpesvirus genomes are required for origin dependent DNA synthesis, including an origin binding protein, a helicase, a primase, a primase-associated factor, a single-stranded DNA binding protein, a DNA polymerase, and a DNA polymerase processivity factor, see for example [1]. In order for the DNA polymerase to copy the viral genome during virus replication, it requires the processivity factor, which binds the polymerase and enhances its ability to synthesize full-length products [2,3]. There is considerable interest in understanding the biology of the polymerase-processivity factor interaction due to the potential for developing herpesvirus-specific drugs that could inhibit genome replication and the production of progeny virus [4,5]. A concerted effort has been made to develop serological reagents that specifically react with each of the different herpesvirus processivity factors to study their cellular localization and trafficking and interactions with other cellular proteins that are critical for virus replication[6-8]. Because of its role in viral DNA synthesis, the processivity factor has also become an important protein marker for virus replication.

Kaposi's sarcoma-associated herpesvirus (KSHV)/human herpesvirus 8, the recently discovered human rhadinovirus belonging to the gammaherpesvirus subfamily, encodes a highly conserved DNA polymerase in open reading frame (ORF) 9 and a DNA polymerase processivity factor in ORF59 [9]. KSHV ORF59 binds DNA as a homodimer, interacts with the ORF9 DNA polymerase to strongly enhance its ability to synthesize full-length DNA [6,10,11] and is necessary for origin-dependent viral DNA replication [12]. In comparison to other human herpesviruses, the KSHV processivity factor displays the strongest similarity (29% amino acid identity) with the processivity factor (BMRF1) of Epstein-Barr virus (EBV), the only other known human gammaherpesvirus. More closely-related processivity factors are present in the related New World and Old World primate rhadinoviruses, herpesvirus saimiri (HVS) of the squirrel monkey (30% identity)[13]) and rhesus rhadinovirus of the rhesus macaque (49% identity) [14,15], respectively. The specificity of the interaction between KSHV ORF59 and its DNA polymerase was demonstrated by the inability of the related processivity factors of herpes simplex type 1 (UL42)(20% identity) and p41 of human herpesvirus 6 (19% identity) to functionally replace KSHV ORF59 [10].

KSHV is now considered to be the cause of Kaposi's sarcoma (KS), pleural effusion lymphoma (PEL) and multicentric Castleman's disease [16]. Essentially all affected cells in these diseases are latently infected with KSHV and only rare cells have active virus replication [17]. Similarly, infection of cells *in vitro *by KSHV results in the rapid establishment of latency with only rare cells showing productive viral replication. Treatment of latently-infected cells with the phorbol ester, TPA, induces activation of virus replication and production of progeny virus. Using an antibody that recognizes KSHV ORF59, the processivity factor was found to be highly expressed in the nuclei of infected cells that harbor actively replicating virus [18,19].

Immunofluorescence studies have localized KSHV ORF59 and other core replication proteins within the viral DNA replication compartments in the nuclei of infected cells [20]. Deletion analysis of ORF59 has revealed regions critical for binding to the DNA polymerase and double-stranded DNA, as well as a region that enhances the processivity of the DNA polymerase [6]. Transport of the viral DNA polymerase into the nucleus is dependent on ORF59 [20], which contains a nuclear localization signal, and binding domains within ORF59 have been identified that are required for nuclear transport of the polymerase [21]. The reactivity of a monoclonal antibody to KSHV ORF59 with the cognate antigen in the nucleus is an important marker of activation of KSHV replication [6].

Rhadinoviruses closely-related to KSHV have been identified in a variety of Old World primates, including macaques, gorillas and chimpanzees. Cloning, sequencing and functional characterization of these viruses is of interest due to the structural and functional similarities with KSHV and the possibility of developing an animal model of KSHV pathology. These Old World primate rhadinoviruses segregate into two distinct lineages. The RV1 lineage consists of KSHV in humans, retroperitoneal fibromatosis herpesviruses (RFHV) in different species of macaque [22], and closely-related viruses in chimpanzees [23,24], gorillas [24] and African Green monkeys [25]. The RV2 lineage consists of rhesus rhadinovirus (RRV) [26] and related viruses in different species of macaques [22,27,28], chimpanzee [29], baboon [30], gibbons [31] and drills [32]. These data suggest that every Old World primate species, including humans, are host to rhadinoviruses of both RV1 and RV2 lineages, although a human RV2 virus has yet to be identified.

Previous studies have shown that RRV, the RV2 rhadinovirus prototype in the rhesus macaque, produces a permissive, replicative infection in cultured rhesus monkey fibroblast cells with obvious cytopathic effects, first evident between 4-7 days, and the production of infectious virus [26]. A genome-wide transcription profile and a more restricted profile of several key RRV genes have been determined during the time course of infection [33,34]. In general, these studies have shown that the transcription of RRV genes after *de novo *permissive infection parallels the transcription profile of KSHV genes after activation of latently-infected cells by treatment with phorbol esters or sodium butyrate.

In order to examine the conservation of ORF59 within the RV1 and RV2 rhadinovirus lineages and develop reagents to detect and differentiate RV1 and RV2 permissive infections, we cloned and sequenced the ORF59 homologs of the RV1 and RV2 rhadinoviruses from chimpanzee and three species of macaque and compared these with the KSHV and RRV ORF59 sequences. Sequence comparisons revealed strong conservation between the RV1 and RV2 rhadinoviruses within the majority of the ORF59 coding sequences. However, lineage-specific sequences were identified in the C-terminal domains, and a polyclonal rabbit antiserum was developed against this domain in the RV2 ORF59 proteins. Specificity of this antiserum was demonstrated by Western blot and immunofluorescence analysis. Using this antiserum and ORF59-specific RT-qPCR assays, we demonstrate that RRV and the related RV2 rhadinovirus, MneRV2, from *M. nemestrina*, both undergo a permissive infection in rhesus primary fetal fibroblast (RPFF) cell cultures and in Vero African green monkey kidney epithelial cells with abundant expression and nuclear localization of ORF59. We also show that RV2 ORF59 expression is coupled with active replication of the viral genome and production of infectious virions. Finally, we demonstrate reactivity of the anti-RV2 ORF59 antiserum within the nuclei of epithelial cells present in the differentiated layer of skin epithelium of a pig-tailed macaque that had been naturally infected with MneRV2. This *in vivo *data with a macaque RV2 rhadinovirus is congruent with previous *in vitro *studies, in which expression of ORF59 and other markers of KSHV replication correlated with epithelial cell differentiation, suggesting that differentiated epithelial cells are a specific source of infectious virions in hosts naturally infected with Old World primate rhadinoviruses.

## Results

### The ORF59 proteins of the Old World primate RV1 and RV2 rhadinoviruses are highly conserved and show lineage-specific conservation in their C-terminal domains

**Figure 1:**
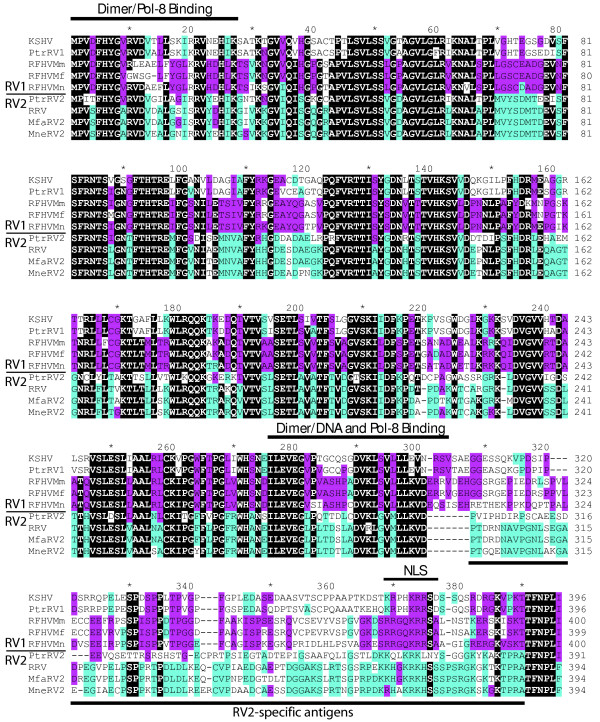
**Comparison of the ORF59 homologs of Old World primate RV1 and RV2 rhadinoviruses**. The *orf59 *genes from the RV1 rhadinoviruses from chimpanzee (PtrRV1) and three species of macaque, *M. mulatta *(RFHVMm), *M. nemestrina *(RFHVMn) and *M. fascicularis *(RFHVMf), and the ORF59 genes from the RV2 rhadinoviruses from chimpanzee (PtrRV2) and the two species of macaque, *M. nemestrina *(MneRV2) and *M. fascicularis *(MfaRV2), were cloned using a CODEHOP PCR approach (see Materials and Methods) and the encoded amino acid sequences were aligned with the previously published sequences of the human RV1 rhadinovirus, KSHV (NP_572115) and the rhesus macaque RV2 prototype, RRV17577 (AAD21393). Identical residues in six of the nine sequences are highlighted in black. Identical residues in at least three of the four RV2 sequences are highlighted in blue, whereas identical residues in at least three of the RV1 sequences are highlighted in purple. Domains involved in nuclear localization (NLS), dimer formation, DNA-binding and DNA polymerase (Pol-8) binding [6,11,21] of KSHV ORF59 are indicated. The RV2-specific antigens of RRV and MneRV2 ORF59 used to produce the anti-RV2 ORF59 rabbit polyclonal antisera are underlined (see Materials and Methods).

We cloned and sequenced the ORF59 homologs from members of the RV1 lineage of Old World primate rhadinoviruses from chimpanzee, *P. troglodyte *(PtrRV1), and three species of macaque, *M. mulatta *(RFHVMm), *M. nemestrina *(RFHVMn) and *M. fascicularis *(RFHVMf), using primers and templates listed in Table 1, as described in Materials and Methods. We also cloned and sequenced the ORF59 homologs from members of the RV2 lineage of Old World primate rhadinoviruses from chimpanzee (PtRV2) and two species of macaque, *M. nemestrina *(MneRV2) and *M. fascicularis *(MfaRV2). We compared these sequences with the previously published sequences of KSHV ORF59 (RV1 lineage in humans) [9] and the ORF59 homolog of the RV2 rhadinovirus from *M. mulatta*, RRV [14]. Alignment of the ORF59 protein sequences revealed strong amino acid sequence conservation with approximately 50% of the residues being identical in all of the sequences from the RV1 and RV2 lineages (highlighted in black, Fig. 1). This strong conservation extended through most of ORF59, up to ~aa300 (KSHV numbering). The C-terminal domains showed little overall conservation between the RV1 and RV2 lineages. Instead, a strong lineage-specific conservation was observed, especially within the macaque RV2 rhadinoviruses extending from aa310-394 (RRV numbering, highlighted in blue, Fig. 1).

**Table 1:** CODEHOP and gene-specific primers for cloning and expression of RV1 and RV2 rhadinovirus ORF59 homologs

Virus/[Sequence source]^1^	Host Species/[DNA source]	Primers (gene)^2^	Primer Sequence (5'-3')
**RV1 Lineage**			

**KSHV** [U93872]	Human (*H. sapiens*) [BCBL cells]	KSHV ORF59a^3^ KSHV ORF59b^3^	GTACAAGGATCCCCTGTGGATTTTCACTATGG ACAGATAAGCTTAAATCAGGGGGTTAAATGTGG

**PtrRV1** (also known as PanRHV1/PtRV1) [this study]	Chimpanzee *(P. troglodyte)* [Ptr001]	SRDEa (ORF60)^4^ PQFVb (ORF59)^4^ IGNGa (ORF59)^5^ PtrRV1 ORF59a^3^ PtrRV1 ORF59b^3^	CTGGCTAACGACTACATCTCCAGRGAYGARCT CCGTAAGAAATGGTGGTCCTGACRAAYTGNGG GAATACTTCCATCGGTAACG TATATAAGATCTAAATCAGTGGGTTAAATGTGG ATTATAAGATCTCCTGTGGATTTTCACTATGG

**RFHVMn** [this study]	Pig-tailed macaque *(M. nemestrina)* [Mne442N]	NFFEa (ORF60)^5^ PQFVb (ORF59)^4^ YGVRb (ORF59)^5^ WCFIb (ORF58)^4^ RFHVMn ORF59a^3^ RFHVMn ORF59b^3^	GGCAGTTTCAAGGCTGTGAATTTTTTTGAGCG CCGTAAGAAATGGTGGTCCTGACRAAYTGNGG CGTCCACCCTGACCCCATA CGAGTACAGGGCCTTGAAGATRAARCACCA GTACAAGGATCCCCTGTGGATTTTCATTATGG GAACTGAAGCTTTTAAATTAATGGGTTAAACG

**RFHVMm** [this study]	Rhesus macaque *(M. mulatta)* [MmuYN91]	NFFEa (ORF60)^5^ PQFVb (ORF59)^4^ FTHTa (ORF59)^5^ YYELb (ORF58)^4^ RFHVMn ORF59a^3^ RFHVMm ORF59b^3^	GGCAGTTTCAAGGCTGTGAATTTTTTTGAGCG CCGTAAGAAATGGTGGTCCTGACRAAYTGNGG CAACGGATTCACGCACACG AAATGCTCCGCAGAAGCCCAGYTCRTARTA GTACAAGGATCCCCTGTGGATTTTCATTATGG GAACTGAAGCTTTCAAATCAACGGGTTAAAGG

**RFHVMf** [this study]	Cynomolgus macaque *(M. fascicularis)* [Mfa95044]	EVEGa (ORF59)^4^ HRYYb (ORF58)^4^ NAAKb (ORF59)^5^ MPVDa (ORF60)^5^ YYELb (ORF58)^4^	TGGCACTCCAACGAAATATTAGARGTNGARGG TGCTAAAAATCCAAGTTCGTARTAYCTRTG CTTCGCAGCATTCCAGGAC ATCATGCCTGTGGATTTTCA AAATGCTCCGCAGAAGCCCAGYTCRTARTA

**RV2 Lineage**			

**PtrRV2** (also known as PanRHV2) [this study]	Chimpanzee *(P. troglodyte)* [Ptr001]	LYNTa (ORF60)^4^ EMFGb (ORF59)^5^ TREMa (ORF59)^5^ GTYTb ORF58)^4^ PtrRV2 ORF59a^3^ PtrRV2 ORF59b^3^	GGCCGCCGGCATGCTGTACAAYACNATGAT TACCCGTGAGATGTTTGGAG CCCATACCAGAGAAATGTTC GAGGGACGCCTCCGACGTGTNCGTNCCCAT ATCATAGATCTCCTATCACATTTCACTACGGAG ATATATAAGCTTAAATAAGGGGATTAAATGTAG

**MneRV2** (also known as PRV/MGVMn) [this study]	Pig-tailed macaque *(M. nemestrina)* [Mne442N]	RDELa (ORF60)^4^ PQFVb (ORF59)^4^ EMFGa (ORF59)^5^ CFICb (ORF58)^4^ MneRV2 ORF59a^3^ MneRV2 ORF59b^3^ T1b (ORF59)^6^ T2b (ORF59)^6^ T3b (ORF59)^6^ T4b (ORF59)^6^ T5a (ORF59)^7^ T6a (ORF59)^7^	CTTGCCAACGATTACATTTCCAGRGAYGARCT CCGTAAGAAATGGTGGTCCTGACRAAYTGNGG TACCCGTGAGATGTTTGGAG TACAAAATACAGCGAGTGATANATRAARCA GTACAAGGATCCCCGGTCTCGTTCCACTACG TAACTGAAGCTTCTAAAACAGCGGGTTGAAGG CTAATTAAGCTTCTAAACTCCAAACATCTCACGGG CTAATTAAGCTTCTAGGTAAACGTGGCAACGGC CTAATTAAGCTTCTAGCCCAACTTGACGTCAGC CTAATTAAGCTTCTACGTTCGCGGTGATTTGGC GTACAAGGATCCCCTACCGGCCAGGAGAATG GTACAAGGATCCAAATCACCGCGAACGAACG

**RRV 17577** [NC_003401]	Rhesus macaque *(M. mulatta)*	RRV ORF59a^3^ RRV ORF59b^3^	GTACAAGGATCCCCTGTCTCGTTTCATTACGG GAACTGAAGCTTAAAACAACGGGTTGAACG

**MfaRV2** (also known as MGVMf) [this study]	Cynomolgus macaque *(M. fascicularis)* [Mfa98044]	RDELa (ORF60)^4^ NRb (ORF59)^5^ NRa (ORF59)^5^ CFICb (ORF58)^4^ MfaRV2 ORF59a^3^ MfaRV2 ORF59b^3^	CTTGCCAACGATTACATTTCCAGRGAYGARCT GGCCCGGAAAATGAGTAACA TCTGAATATGTCACATCCGTTCATA TACAAAATACAGCGAGTGATANATRAARCA GTACAAGGATCCCCTGTCTCGTTTCATTACGG TAACTGAAGCTTCTAAAACAGCGGGTTGAACG

^1 ^We have utilized the virus nomenclature scheme indicating the host genus (first letter), species (second and third letters) with the designation of rhadinovirus lineage, ie RV1 or RV2, to provide a unique designation for the rhadinoviruses from different Old World primate species. For historical reasons, Homo sapiens HsaRV1 remains as KSHV, the macaque RV1s remain as RFHVMn, RFHVMm, and RFHVMf and the rhesus macaque MmuRV2 remains as RRV. The gene-specific primers are derived from published sequences (Genbank accession number is indicated) or from new sequences obtained in this study.

^2 ^a and b designations indicate forward and reverse strand primers, respectively

^3 ^5' and 3' gene-specific primers used for preparing the full-length ORF59 bacterial expression vectors

^4 ^CODEHOP PCR primers used for cloning the initial regions of the ORF59 genes

^5 ^gene-specific primers used in conjunction with the CODEHOP PCR primers to obtain the full-length ORF59 coding sequences

^6 ^gene-specific primers used in conjunction with the MneRV2 ORF59a primer to construct MneRV2 ORF59 truncation mutants

^7 ^gene-specific primers used in conjunction with the MneRV2 ORF59b primer to construct MneRV2 ORF59 truncation mutants

Overall, the chimpanzee RV1 rhadinovirus (PtrRV1) ORF59 was most closely related to the KSHV ORF59 sequence with 89% identical amino acids (Table 2). While the similarity of the macaque RV1 sequences to both the PtrRV1 and KSHV sequences ranged from 56-58%, the similarity between macaque RV1 sequences themselves was 82-94%. Within the RV2 lineage, the ORF59 sequences of the different macaque rhadinoviruses were 88-95% conserved with each other and 64% conserved with the PtrRV2 chimpanzee sequence. The similarity of the KSHV ORF59 and RV2 ORF59 sequences ranged from 49-51% (see Table 2).

**Table 2:** Amino acid sequence comparisons of the ORF59 homologs of human, macaque and chimpanzee RV1 and RV2 rhadinoviruses

	MneRV2	RRV	MfaRV2	PtrRV2	RFHVMn	RFHVMm	RFHVMf	PtrRV1
**RRV**	88%							

**MfaRV2**	89%	95%						

**PtrRV2**	64%	65%	65%					

**RFHVMn**	52%	50%	51%	49%				

**RFHVMm**	49%	49%	49%	47%	82%			

**RFHVMf**	50%	50%	50%	48%	82%	94%		

**PtrRV1**	51%	51%	50%	50%	58%	57%	56%	

**KSHV**	51%	49%	49%	49%	58%	56%	57%	89%

Alignment of the ORF59 sequences from the RV1 and RV2 Old World primate rhadinoviruses revealed strong sequence conservation of several functional domains that have been identified in KSHV ORF59. Dimerization and binding domains for the KSHV DNA polymerase have been identified within the KSHV ORF59 sequence at aa1-27 and aa276-304 (see Fig. 1). These regions showed blocks of strong conservation across all ORF59 sequences interspersed with blocks conserved between the different rhadinovirus lineages. A three amino acid motif "KRR" (aa373-375, KSHV) adjacent to a serine residue at position aa376 in the KSHV ORF59 was found to be critical for nuclear localization of both ORF59 and the DNA polymerase of KSHV [21]. This motif and the downstream serine were conserved in all of the RV1 ORF59 sequences analyzed (Fig. 1). A similar motif "KRK" (aa369-371, RRV) was conserved in all the RV2 ORF59 sequences analyzed. This motif was separated by one amino acid from a serine residue which was conserved in all three macaque RV2 OR59 sequences but not the chimpanzee RV2 ORF59.

**Figure 2:**
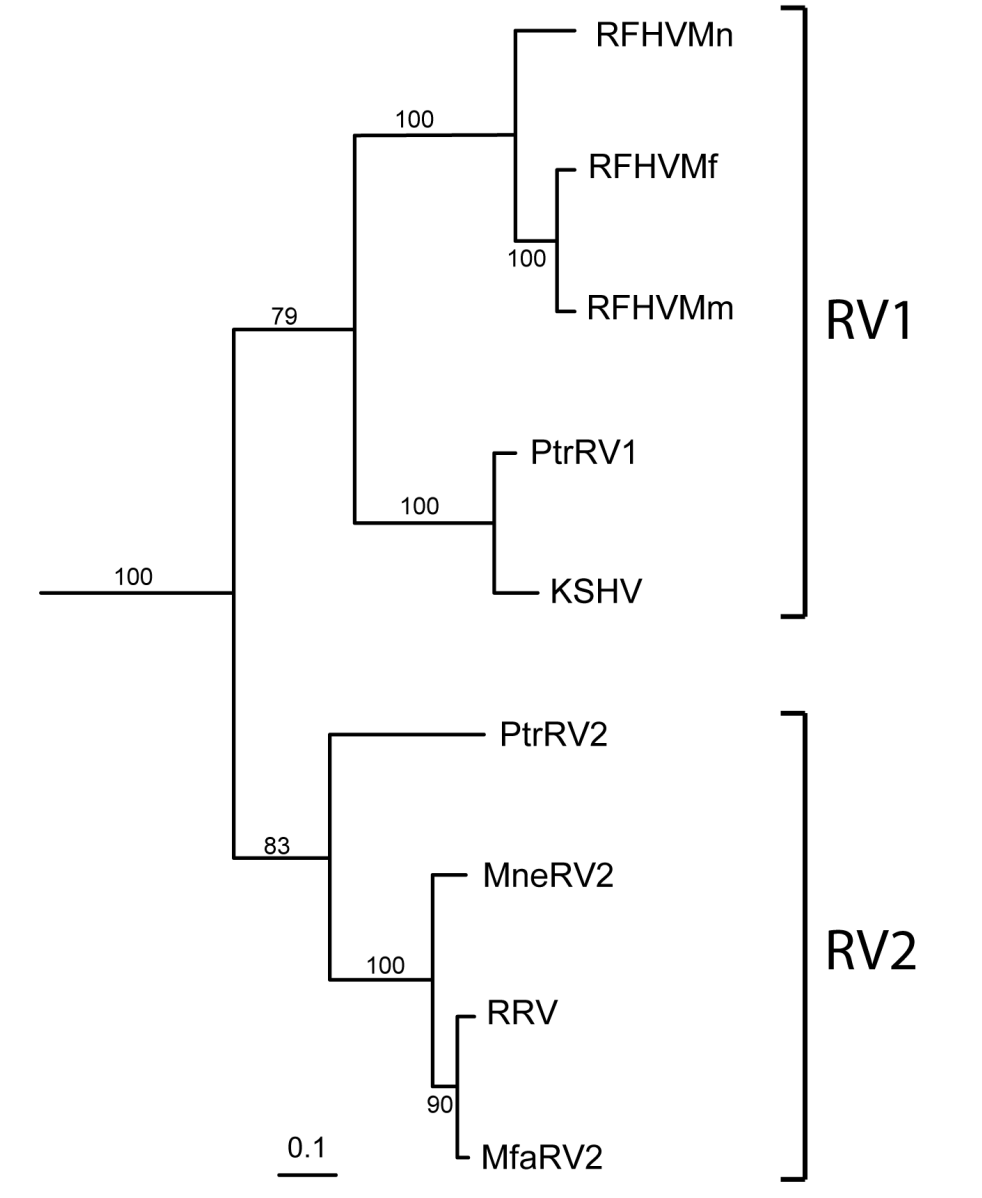
**Phylogenetic analysis of the RV1 and RV2 ORF59 protein sequences (Fig. 1) using protein maximum-likelihood**. The ORF59 homolog of the New World primate rhadinovirus, herpesvirus saimiri (NP_040261), was used as an outgroup. Bootstrap values for 100 replicate samplings and the scale for substitutions per site are provided.

Phylogenetic analysis of the aligned amino acid sequences revealed a separate clustering of the RV1 and RV2 ORF59 sequences, with KSHV ORF59 clustering with the RV1 sequences (Fig. 2), as expected from previous studies with other viral genes [22]. In the RV1 cluster, the ORF59 sequences from the human (KSHV) and chimpanzee (PtrRV1) viruses grouped together, demonstrating a close evolutionary relationship. The ORF59 sequences from the macaque RV1 rhadinoviruses clustered together with the sequences of the *M. mulatta *and the *M. fascicularis *rhadinoviruses, RFHVMm and RFHVMf, respectively, showing the closest similarity. ORF59 from the *M. nemestrina *rhadinovirus, RFHVMn, was an outgroup of the macaque virus sequences. The chimpanzee and macaque RV2 ORF59 sequences clustered separately from KSHV and the other RV1 ORF59 sequences, with the chimpanzee sequence as an outgroup of the macaque sequences. Within the macaque RV2 rhadinoviruses, the ORF59 sequences from *M. mulatta *(RRV) and *M. fascicularis*, (MfaRV2) clustered together, with the ORF59 from *M. nemestrina *(MneRV2) as an outgroup.

### RRV *orf59 *is transcribed early after RRV infection of RPFF cells

**Figure 3:**
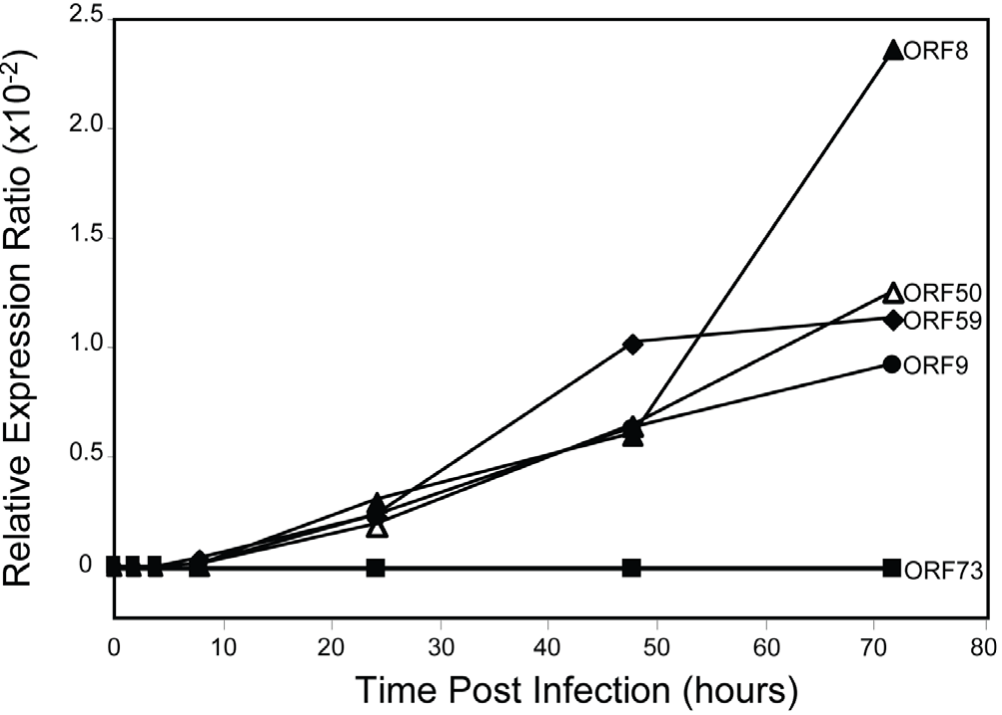
**RRV ORF59 mRNA expression after RRV infection of RPFF cells**. Near confluent cultures of RPFF cells were infected with RRV and incubated for various times. Levels of mRNA expressed by the RRV genes *orf59 *DNA polymerase processivity factor, *orf50 *transactivator, *orf8 *glycoprotein B, *orf73 *latency-associated nuclear antigen and *orf9 *DNA polymerase were determined by RT-qPCR as described in Materials and Methods. Viral mRNA levels were normalized to mRNA levels of the cellular ribosomal phosphoprotein (RPO) and expressed as a relative expression ratio.

The *orf59 *of the RV1 rhadinovirus, KSHV, has been classified as an early-late gene due to its low level expression in latently-infected cells and increased expression after activation of latently-infected cells to begin replicating viral DNA and producing infectious virions [18]. In order to study the expression kinetics of the *orf59 *of an RV2 rhadinovirus, rhesus primary fetal fibroblast (RPFF) cell cultures were infected with RRV and total nucleic acids were extracted at different times after infection. The mRNA levels of RRV *orf59 *were compared to those of the RRV homologs of the *orf50 *lytic transactivator gene, the *orf9 *DNA polymerase, the *orf8 *glycoprotein B and the *orf73 *latency-associated nuclear antigen (LANA) using gene-specific RT-qPCR assays to quantitate mRNA, as described in the Materials and Methods. Viral mRNA expression levels were normalized to the mRNA levels of the cellular ribosomal phosphoprotein gene (RPO). RRV *orf59 *mRNA was first detected 8 hours post infection and its levels continued to rise strongly until 48 hours post infection. Subsequently, the levels remained fairly constant through 72 hours post infection. (Fig. 3). Similarly, mRNAs for the RRV *orf8*, *orf9*, and *orf50 *were first detected at 8 hours post infection. The levels of these mRNAs continued to rise over the 72 hour time course with a strong increase in the *orf8 *glycoprotein B mRNA by 72 hours. A very low level of ORF73 LANA mRNA was reproducibly detected 8 hours post infection, but no increase was observed over the 72 hour time course. Maximal mRNA copy number for ORF59 reached ~32,800, while the copy numbers for ORF73, ORF50, ORF9 and ORF8 reached ~50, 20,900, 20,200 and 25,700, respectively.

### ORF59 homologs elicit a strong humoral antibody response in macaques naturally infected with RV1 or RV2 rhadinoviruses in vivo

**Figure 4:**
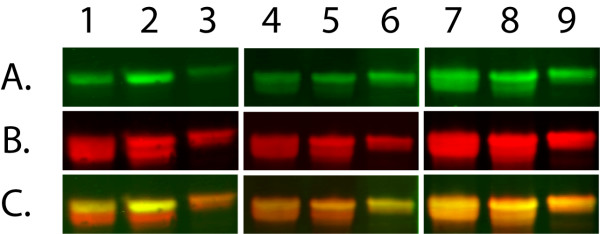
**Naturally infected- macaques develop strong humoral immune responses against RV1 and RV2 ORF59 homologs**. 6Xhis-ORF59 fusion proteins from MneRV2 (lanes 1,4 and 7), RRV (lanes 2,5 and 8) and RFHVMn (lanes 3,6 and 9) were electrophoresed and transferred to membranes. The blots were first probed with 1:1,000 dilution of macaque plasma from either dBL2 (adult, non-SIVinfected rhesus macaque; lanes 1-3), J00079 (adult SIV-infected pig-tailed macaque; lanes 4-6) and K99344 (adult SIV-infected pit-tailed macaque; lanes 7-9) with a secondary Dylight 700 anti-human IgG antibody (green, Row A). The blots were washed and the levels of recombinant protein were quantitated using an anti-6XHis mouse monoclonal antibody and a secondary Dylight 800 anti-mouse IgG antibody (red, Row B). The overlay of red and green staining (yellow) is shown in Row C.

Previous studies have shown that ORF59 of KSHV is immunogenic in KSHV-infected patients with KS [35] and serological assays have been developed to detect antibodies to KSHV ORF59 to study virus prevalence and detect infection [36]. To determine whether the macaque RV1 and RV2 ORF59 proteins are immunogenic in naturally-infected macaques, full length ORF59 proteins from RRV, MneRV2, and RFHVMn were expressed as 6XHis-fusions in the pQE30 vector. The 6XHis-ORF59 fusion proteins were purified on Ni-NTA agarose and analyzed by immunoblotting. Significant amounts of 6XHis-ORF59 fusion proteins were detected in each case using an anti-6XHis antibody with molecular weights ranging from 44-45 Kd (Fig. 4, RowB). The immunoblot was probed with plasma from several juvenile pig-tailed macaques (age ~1 year). No reactivity was detected (data not shown). Various immunoreactivities were detected with plasma from older macaques and macaques infected with SIV. Serum from the adult rhesus macaque dBL2 reacted strongly with the full-length RRV ORF59 protein (Fig. 4, lane2A, C), and more weakly with the MneRV2 and RFHVMn ORF59 proteins (Fig. 4, lanes1A, C and 3A, C, respectively). Serum from the SIV-infected pig-tailed adult macaque J00079 reacted most strongly with the RFHVMn ORF59 (Fig. 4, lane 6A, C), with weaker reactivity to both the MneRV2 and RRV ORF59 (Fig. 4, lanes 4A, C and 5A, C, respectively). In contrast, serum from the SIV-infected macaque K99344 reacted strongly with all three macaque RV1 and RV2 ORF59 proteins (Fig. 4, lanes 7-9A, C).

The reactivity of the different macaque sera to ORF59 was examined by Western blot analysis of N- and C-terminal truncations of the MneRV2 ORF59, as described above. All three macaque sera exhibited strong reactivity to the T1 C-terminal truncation mutant containing only 101 N-terminal amino acids of the 394 amino acid MneRV2 ORF59 (Table 3). Sera reactivity was also seen to the T2, T3 and T4 C-terminal truncation mutants which also contained the N-terminal amino acids of the T1 truncation. No sera reactivity was detected to the T5 and T6 N-terminal truncation mutants containing only the C-terminal amino acids 299-394 or 354-394, respectively (Table 3). These results indicate that at least the first 101 amino acids and maybe more of the highly conserved ORF59 N-terminal domain contain antigenic epitopes recognized by the macaque immune system during natural rhadinovirus infections.

**Table 3:** Epitope mapping of MneRV2 ORF59 using N- and C-terminal truncation mutants.

MneRV2 ORF59 truncation^1^	N-terminal residue^2^	C-terminal residue^2^	Macaque serum^3^		
			
			DBL2	J00072	K99344
**T1**	1	101	+	+	+

**T2**	1	205	+	+	+

**T3**	1	291	+	+	+

**T4**	1	360	+	+	+

**T5**	299	394	-	-	-

**T6**	354	394	-	-	-

^**1 **^*orf59 *gene truncations were prepared using primers listed in Table 1 and truncated proteins were expressed in bacteria as described in Materials and Methods.

^**2 **^Amino acid residue number from the sequence of MneRV2 ORF59 (Fig. 1)

^**3 **^Serum (1:1000 dilution) from SIV-negative rhesus macaque (DBL2) and SIV-positive pig-tailed macaques (J00072 and K99344) were reacted with MneRV2 ORF59 truncation mutants in Western blots as described in Figure 4 and the Materials and Methods.

### Development of a pan anti-macaque RV2 ORF59 rabbit polyclonal antiserum

**Figure 5:**
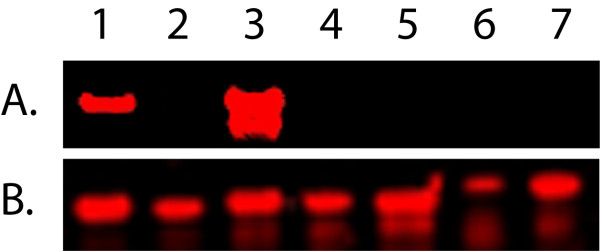
**The 425 rabbit anti-RV2 ORF59 antiserum specifically recognizes the ORF59 proteins from the RV2 rhadinoviruses of three macaque species**. Recombinant C-terminal polypeptides that were highly conserved between RRV and MneRV2 (see underlined antigenic sequences in Fig. 1) were expressed in bacteria with a 6XHis tag, purified and used to immunize a rabbit (425). Full length recombinant ORF59 proteins from RRV (Lane 1), RFHVMm (Lane 2), MneRV2 (Lane 3), RFHVMn (Lane 4), PtrRV2 (Lane 5), PtrRV1 (Lane 6) and KSHV (Lane 7) were expressed as glutathione synthase tranferase (GST) fusions, analyzed by SDS-PAGE and probed using either A) 425 rabbit anti-RV2 ORF59 antiserum, or B) anti-GST antiserum, as described in the Materials and Methods.

To study the function and expression of the RV2 ORF59 proteins, we developed rabbit polyclonal antisera recognizing the ORF59 of several macaque RV2 rhadinoviruses. Alignment of the ORF59 sequences from the RV1 and RV2 macaque rhadinoviruses revealed a strong conservation between RV2 sequences within the ORF59 carboxy-terminal region (Fig. 1). Since the region from amino acid 300-388 of the RV2 sequences showed little conservation with the RV1 ORF59 sequences, this region was chosen as an antigenic target for the development of specific anti-macaque RV2 ORF59 antisera. The coding regions for these 89 amino acids from both RRV and MneRV2 ORF59 were cloned into the 6XHis expression vector, pQE30, and recombinant proteins were produced in bacteria and purified, as described in Materials and Methods. Equal amounts of the purified RRV and MneRV2 ORF59 polypeptides were combined and used to immunize rabbits. The 425 rabbit anti-RV2 ORF59 antiserum showed strong reactivity with the RV2 ORF59 proteins from both RRV and MneRV2 (Fig. 5) but not with ORF59 from the chimpanzee PtrRV2. This absence of cross-reactivity correlated with lack of conservation within the antigenic region between the macaque and chimpanzee RV2 sequences (see Fig. 1, aa300-388, RRV numbering). The anti-RV2 ORF59 antiserum also did not react with the ORF59 proteins from the RV1 lineage rhadinoviruses, including RFHVMn, RFHVMm, PtrRV1 and KSHV (Fig. 5), nor with the ORF59 homolog (BMRF1) of EBV (data not shown) which shares a nearly identical C-terminal domain with the BMRF1 homologs of the macaque lymphocryptoviruses.

### RV2 ORF59 proteins are highly expressed in the nuclei of infected fibroblast and epithelial cells *in vitro*

**Figure 6:**
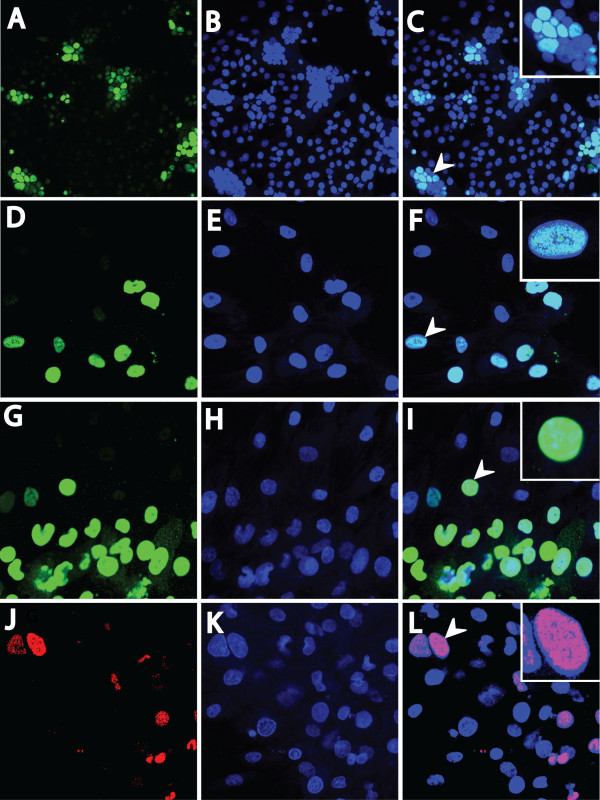
**The RV2 ORF59 proteins are highly expressed during RV2 rhadinovirus infections of RPFF and Vero cells and localize to the nucleus**. Subconfluent cell cultures were infected with either RRV or MneRV2, and ORF59 expression was detected by confocal immunofluorescence microscopy using the anti-RV2 ORF59 antiserum. Nuclear DNA was visualized with Topro-3 stain. ORF59 immunofluorescence (A, D, G, and J), Topro-3 nuclear fluorescence (B, E, H and K), and an overlay of ORF59 and Topro-3 fluorescence (C, F, I, and L) are shown. (A-C) Vero cells infected with RRV and reacted with the rabbit 425 anti-RV2-ORF59 antiserum (10× magnification). (D-F) RPFF cells infected with RRV and reacted with the 425 antiserum (40× magnification). (G-I) RPFF cells infected with MneRV2 and reacted with 425 antiserum (40× magnification). (J-L) RPFF cells infected with MneRV2 and reacted with mouse anti-HHV8 ORF59 monoclonal antibody (40× magnification). Arrows indicate the cell/s shown in the inserts with evidence of syncytia/aggregation (C), and co-localization of RV2 ORF59 and nuclear DNA (F, I and L).

**Figure 7:**
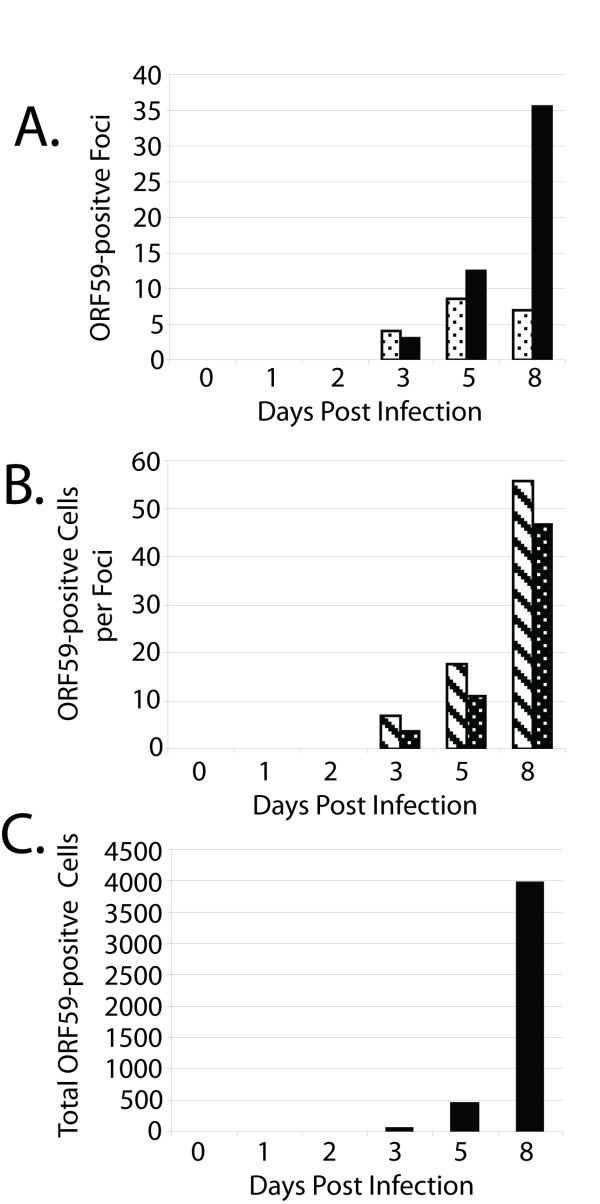
**ORF59 is a marker for RRV infection and provides evidence for efficient virus transmission in local environments**. Subconfluent cultures of RPFF cells were infected with RRV (~MOI 0.01) for 2 hours. Input virus was removed and cells were washed, incubated for various times, fixed and reacted with the 425 anti-RV2 ORF59 antiserum. ORF59 expression was visualized by confocal immunofluorescence microscopy and fluorescent nuclei were manually counted. Single cell and multi-cell foci of infection were differentiated (see visual examples in Fig. 6A, C). A) singe cell foci - dotted bar; multi-cell foci - solid bar B) cells per multi-cell foci - dashed bar; cells per all foci - dotted bar.

The RV1 ORF59 from KSHV accumulates in the nuclei of latently-infected cells *in vitro *after activation by TPA or sodium butyrate treatment to initiate viral replication [18,19]. To determine the localization of the RV2 ORF59 proteins, semi-confluent cultures of fibroblast cells (RPFF) or epithelial cells (Vero) were infected with purified RRV at an MOI of ~0.01. The infected cell cultures were incubated for 0, 1, 2, 3, 5 or 8 days, fixed, incubated with the rabbit anti-RV2 ORF59 antiserum and examined by confocal microscopy. Numerous foci of ORF59-positive cells were detected in both the RRV-infected Vero cells (Fig. 6A-C; 10× magnification - obvious single and multi-cell foci with fluorescent nuclei are present) and RPFF cells (Fig. 6D-F; 40× magnification of a multi-cell focus of infection). In the infected RPFF cells, seven ORF59-positive foci of infection were detected at day 3 (four single cell foci and 3 multi-cell foci)(Fig. 7A). This increased to a total of 43 ORF59-positive foci by day 8 (seven single cell foci and 36 multi-cell foci). At day 3, the multi-cell foci contained on average approximately seven ORF59-positive cells per foci (Fig. 7B). By day 8, this had increased to an average of 56 ORF59-positive cells per foci. The total number of ORF59-positive cells increased from 49 (day 3) to 457 (day 5) and to 3974 (day 8) (Fig. 7C). No obvious ORF59-positive cells were detected prior to day 3.

The ORF59-positive foci of infected Vero cells showed a distinct clustering suggesting syncytia formation seen with other herpesviruses (Fig. 6A-C). This clustering/aggregration is more obvious in the image of the ToPro-3-stained cell nuclei (Fig. 6B). These ORF59-positive clusters contained strongly positive cells, weakly positive cells and cells with no apparent ORF59 staining (see inset, Fig. 6C). The ORF59-positive foci of infected RPFF cells showed no obvious clustering or aggregation (Fig. 6D-F). Examination of the ORF59-positive foci in the RPFF cells at high magnification (40×) revealed a co-localization of ORF59 immunofluorescence (green) and Topro-3 DNA fluorescence (blue) consistent with a nuclear localization of ORF59 (see insert, Fig. 6F). The ORF59 staining was widespread throughout the nucleus, but had a speckled or mottled appearance revealing distinct areas with minimal ORF59. No antibody reactivity was seen with cells that were mock-infected (data not shown), and a clear demarcation of the ORF59-positive cells and negative cells was observed in the periphery of the infection foci showing specificity of the antibody reaction (Fig. 6A-F).

A similar analysis of RPFF cells infected with the closely-related RV2 rhadinovirus from pig-tailed macaques, MneRV2, was performed. Confocal analysis of the MneRV2-infected RPFF cells revealed numerous foci reacting with the RV2 ORF59 antiserum (Fig. 6G-I). The rabbit anti-RV2 ORF59 antisera gave strong fluorescence that co-localized with Topro-3 within the nucleus. (Fig. 6I and insert). With both the RRV and MneRV2 infections, an obvious gradient of ORF59 immunofluorescence was seen within the positive foci. Strong fluorescence was observed in the cells within the center of the positive foci (Fig. 6). Weaker fluorescence was observed in the cells at the edge of the foci, suggesting that the virus infection was permissive and was spreading from an epicenter by production of new infectious virions and infection of adjacent cells. These results demonstrate that the rabbit anti-RV2 ORF59 antisera reacts with the ORF59 proteins expressed *in vitro *by both RRV and MneRV2. Furthermore, they show that ORF59 is produced and accumulates within the nucleus during these permissive infections.

To examine the specificity of the commercial mouse monoclonal antibody developed against the KSHV ORF59, RPFF cell cultures infected with RRV or MneRV2 were fixed and stained with the mouse monoclonal antibody. While no reactivity was observed with RPFF cells infected with RRV, strong nuclear fluorescence staining was observed in numerous foci in RPFF cells infected with MneRV2 (Fig. 6J-L). To confirm this reactivity, the RV1 and RV2 ORF59 recombinant 6XHis-fusion proteins were examined for reactivity with the anti-KSHV ORF59 monoclonal antibody. While the RFHVMn, and MneRV2 ORF59 proteins both reacted with the monoclonal, the RRV ORF59 protein did not (data not shown).

### RRV infection of RPFF and Vero cells is permissive and results in viral genome replication and production of infectious virions

**Figure 8:**
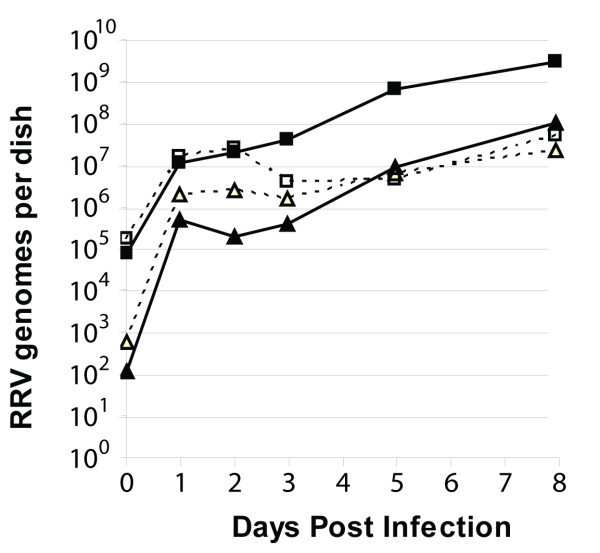
**RRV infection of RPFF and Vero cells is permissive with replication of viral genomes and production of infectious virions**. Near confluent cultures of RPFF (solid lines) and Vero cells (dotted lines) were infected with RRV and incubated for various times. Cell-free (triangles) and cell-associated (squares) RRV DNA was quantitated by TaqMan qPCR from culture medium and cell pellets, respectively. The 0-time point reflects the amount of input virus that bound to the cell monolayer during the adsorption period. For the longer time points, the spent culture medium was collected during the course of the incubation, combined and PEG precipitated before analysis.

In order to examine the relationship between ORF59 expression and genome replication during permissive infections, the time course of RRV genome accumulation was determined after RRV infection of RPFFs. These cells are known to be permissive for RRV infection [14,26]. RRV infection of RPFFs was compared to infection of African green monkey Vero cells, which have previously been reported to be unable to support RRV replication [37]. Subconfluent cultures of RPFF and Vero cells were infected with purified RRV virus at an approximate MOI of 0.01 and the amount of cell-associated RRV DNA and cell-free RRV DNA in the culture supernatant was assayed using an RRV-specific TaqMan qPCR assay [30]. By 24 hours post infection, the cell-associated RRV DNA increased more than 100 fold over the amount associated with the cell monolayer during the virus adsorption period in both the RPFF and Vero cell cultures (Fig. 8). At 24 hours the amount of cell-free RRV DNA present in the culture medium increased by nearly four logs in both cell cultures. By day 8, the cell-associated RRV DNA in the infected RPFF cultures had increased by almost 5 logs over the amount present after the initial adsorption period, while the cell-free RRV DNA had increased by 6 logs. By day 8, the cell-associated and cell-free RRV DNA in the infected Vero cultures had increased by almost 3 logs and 5 logs, respectively.

To determine whether this increase in RRV DNA represented infectious virus, aliquots of the RRV DNA-containing culture medium were obtained 5 days post infection from both infected RPFF and Vero cell cultures and were added to subconfluent cultures of uninfected RPFF cells. After 5 days, the newly infected RPFF cell cultures showed significant cytopathic effects (data not shown). qPCR analysis of cell-associated and cell-free RRV DNA revealed greater than 10^7 ^viral genomes in both the RPFF cultures receiving medium from either the initial RPFF or Vero infected cultures. These results demonstrate that RRV infection of both RPFF and Vero cells is permissive resulting in viral genome replication and production of infectious RRV virions, which correlates with the expression and nuclear accumulation of RRV ORF59.

### Macaque RV2 ORF59 is highly expressed in nuclei of epithelial cells present in the differentiated layer of stratified epithelium in the skin of a naturally infected macaque *in vivo*

**Figure 9:**
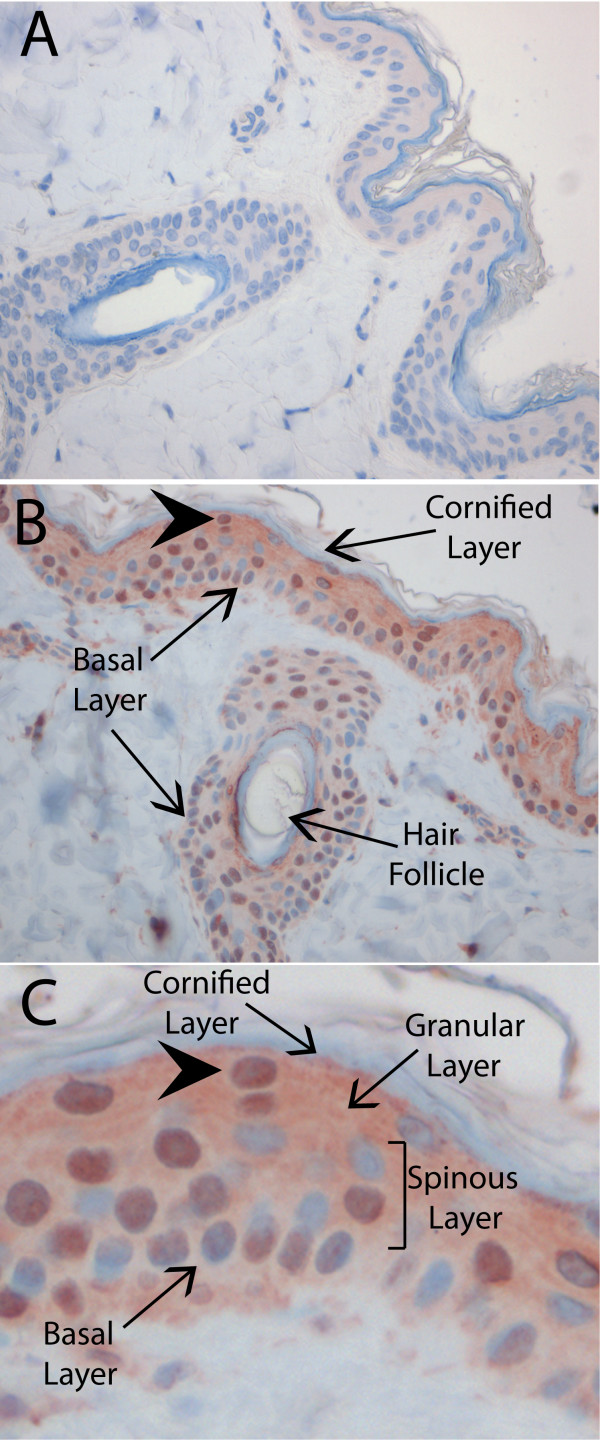
**Expression of RV2 ORF59 in the skin of a pig-tailed macaque naturally infected with the MneRV2 rhadinovirus and simian retrovirus-2 (SRV-2)**. Formalin-fixed skin tissue from (A) a juvenile (MneRV2 and SRV-2 negative) and (B) an adult (MneRV2 and SRV-2 positive) pig-tailed macaque from the WaNPRC was reacted with the 425 rabbit anti-RV2 ORF59 antiserum as described in Materials and Methods. A magnification of a section of the tissue in B (large arrowhead) is shown in (C). The different layers of the epidermal tissue are indicated. The vertically-oriented cells in the basal layer correspond to the undifferentiated, proliferating, columnar basal keratinocytes. The round cells present in the spinous layer and the flattened cells present in the granular layer correspond to the post-mitotic suprabasal keratinocytes.

To investigate the *in vivo *expression of ORF59 in pig-tailed macaques naturally infected with the RV2 rhadinovirus, MneRV2, slides were prepared from formalin-fixed paraffin-embedded skin tissue of a young (1.84 yr old) female *M. nemestrina *that presented with a chronic SRV-2 infection at the WaNPRC. The anti-RV2 ORF59 serum from rabbit 425 displayed no reactivity with stratified skin epithelium from a juvenile macaque negative for both MneRV2 and SRV-2 viruses (Fig. 9A). In contrast, the anti-RV2 ORF59 antiserum strongly reacted with the nuclei of keratinocytes in the stratified epithelium of the skin and in the epithelium surrounding the hair follicles (Fig. 9B). The columnar cells within the basal epithelial layer showed variable antibody reactivity, with strong staining observed in only ~10-12% of the cells. Within the spinous layer, a number of cells were detected that had strong anti-RV2 ORF59 staining in large, round nuclei. Finally, a few strongly stained nuclei were detected in the granular layer immediately below the cornified epithelium (Fig. 9B). These nuclei appeared flattened and were distinct from the round spinous and columnar basal cells. Approximately 50-60% of the suprabasal cells stained strongly for RV2 ORF59. Our results demonstrate that the suprabasal keratinocytes are infected with MneRV2 and express nuclear ORF59 suggesting that they are actively replicating the viral genome and producing infectious virions.

## Discussion

We have compared the ORF59 homologs of members of the RV1 and RV2 lineages of KSHV-like rhadinoviruses from chimpanzee and three species of macaques. We obtained the complete sequences of the ORF59 homologs of the RV1 and RV2 rhadinoviruses from chimpanzee (PtrRV1 and PtrRV2) and the cynomolgus macaque (RFHVMf and MfaRV2). These are the only complete gene sequences presently determined for these rhadinovirus species. We also obtained the complete ORF59 sequences of the RV2 rhadinovirus from pig-tailed macaques (MneRV2) and the RV1 homologs from pig-tailed and rhesus macaques (RFHVMn and RFHVMm). We compared the sequences obtained in the current study with the published sequences of ORF59 from KSHV [9] and the rhesus macaque RRV [14]. Sequence alignment and phylogenetic analysis of the ORF59 sequences from these viruses confirmed the existence of separate RV1 and RV2 lineages of KSHV-like rhadinoviruses in Old World primates, which was originally demonstrated using fragments of the viral DNA polymerase gene [22-25]. The ORF59 sequence comparisons and phylogenetic analyses strongly support a close evolutionary relationship between KSHV and the RV1 rhadinoviruses from the chimpanzee (PtrRV1) and macaque (RFHVMn, RFHVMm, and RFHVMf), while the rhesus macaque rhadinovirus, RRV, and the other RV2 rhadinoviruses constitute a separate and distinct lineage of KSHV-like rhadinoviruses.

Within the RV1 lineage, the ORF59 sequences of the human and chimpanzee rhadinoviruses, KSHV and PtrRV1, respectively, were closely related, reflecting the close evolutionary relationship of human and chimpanzee hosts. No RV2 rhadinovirus has yet been identified in humans to date, so the relationship between the human and chimpanzee RV2 rhadinoviruses is unknown. In both the RV1 and RV2 lineages, the ORF59 sequences from the *M. mulatta *and *M. fascicularis *rhadinoviruses clustered together, with the *M. nemestrina *viral sequences as a distinct outgroup. Phylogenetic studies have shown that the macaque species *M. mulatta *and *M. fascicularis *are more closely related to each other than they are to the *M. nemestrina *species which evolved in a geographically distinct area [38]. Thus, the phylogenetic clustering of the viral ORF59 sequences from the different RV1 and RV2 rhadinoviruses mirrors the phylogenetic relationship of their primate hosts, providing strong evidence for co-speciation of the virus and host.

A comparison of the ORF59 sequences revealed a higher conservation among the RV2 rhadinoviruses than was observed for the RV1 rhadinoviruses from the same primate species, even though the RV1 and RV2 viruses appear to have co-evolved within the same host species over the same period of time. The amino acid conservation between the chimpanzee and macaque RV2 rhadinoviruses was 64-65% whereas the conservation between the chimpanzee and macaque RV1 rhadinoviruses was only 56-58%. Similarly, conservation between rhadinoviruses from *M. nemestrina *and *M. mulatta/M. fascicularis *macaque species was 88-89% for the RV2 lineage and 82% for the RV1 lineage. Finally, conservation between the *M. mulatta *and *M. fascicularis *macaque species was 94.9% for the RV2 lineage and 93.8% for the RV1 lineage. This suggests that the two viral lineages have experienced different evolutionary pressures within the same host that has directly affected the rate of divergence of the ORF59 gene. Functionally, the ORF59 gene family encodes a DNA polymerase processivity factor which is required for copying the viral genome during the replicative cycle of the virus for the production of new virions. Our data suggests that an inherent difference in the biology of the RV1 and RV2 lineages has exerted an effect on the divergence of the ORF59 gene.

Nevertheless, our studies show a strong sequence similarity between the ORF59 sequences of the RV1 and RV2 rhadinoviruses within the N-terminal 300 amino acids with 45% of the amino acids conserved in all of RV1 and RV2 species examined. Our truncation study indicated that the N-terminal domains of the macaque RV2 rhadinovirus ORF59 proteins contain one or more antigenic epitopes recognized in naturally infected macaques. This contrasts with the epitopes of the ORF59 homologs of KSHV and EBV (BMRF1) recognized by mice during development of murine monoclonal antibody reagents, which cluster in the C-terminal ORF59 domain. Although only 6% of amino acid residues in the ~100 amino acid C-terminal domain were conserved across all of the RV1 and RV2 species, a strongly lineage-specific conservation was detected, especially within the macaque RV2 sequences. We developed a rabbit anti-RV2 ORF59 polyclonal antiserum against the conserved C-terminal domain of the RRV and MneRV2 ORF59 proteins. This antiserum reacts specifically with the ORF59 homologs of different macaque RV2 rhadinoviruses and not with ORF59 homologs of RV1 rhadinoviruses or with the related ORF59 homolog (BMRF1) of EBV or macaque lymphocryptoviruses. The antiserum does not react with the ORF59 homolog of the chimpanzee RV2 rhadinovirus due to significant differences in the amino acid sequence of the chimpanzee virus in the targeted C-terminal region.

We tested the immunoreactivity of the commercial monoclonal antibody developed against KSHV ORF59 with the different macaque ORF59 proteins. Recombinant ORF59 protein from the macaque RV1 rhadinovirus, RFHVMn, reacted with the anti-KSHV ORF59 monoclonal antibody. Surprisingly, this antibody also showed reactivity with the MneRV2 ORF59, but not the RRV ORF59. Truncation studies have indicated that residues 279-301 in KSHV ORF59 constitute part of the epitope recognized by anti-ORF59 monoclonal antibody 11D1[6]. Examination of this region in the ORF59 alignment in Figure 1, revealed an amino acid substitution in aa292 from a Lys in KSHV ORF59 to an Arg in RRV ORF59. All other RV1 and RV2 ORF59 sequences contained the Lys residue in this region of high sequence conservation. These sequence comparisons suggest that the Lys at residue 299 may compose part of the monoclonal epitope that is disrupted by the Arg in the RRV ORF59 sequence.

In this study, we used the rabbit anti-RV2 ORF59 antiserum to analyze RV2 rhadinovirus infections of cultured cells *in vitro*. Our results demonstrate that significant numbers of fibroblast (RPFF) and epithelial (Vero) cells express ORF59 after infection with RRV or the closely related RV2 rhadinovirus from pig-tailed macaques, MneRV2. ORF59 expression is localized to the nucleus of infected cells in a non-uniform pattern. Our studies indicate that the RV2 infection in these cells spreads from an initial focus of infection, detected as a single ORF59-positive cell, to adjacent cells that over time also become ORF59 positive. Infection of adjacent cells could be due to cell-cell transmission by passage of cell-associated virus through physical contact, or due to a strong concentration gradient of cell-free virus immediately surrounding the infected ORF59-positive cell that results in preferential infection of immediately adjacent cells. However, numerous cases were identified in our micrographs in which an ORF59-negative cell was detected in close proximity to one or more ORF59 positive cells (see cells immediately adjacent to the labeled cells in panels 5F and I). This suggests that direct physical contact between infected and non-infected cells is more important for virus transmission than proximity. We also continued to detect single ORF59-positive cells during the 8 day incubation, indicative of *de novo *infections in the cell culture by cell-free virus.

KSHV ORF59 is critical for replication of the viral genome within the nucleus [6,10-12]. This processivity factor has an arginine/lysine rich nuclear localization signal in its C-terminal domain that is responsible for targeting the protein to centers of replication in the nucleus [20,21]. We identified homologous arginine/lysine rich domains in both the chimpanzee and macaque RV1 and RV2 ORF59 proteins that could function to target the proteins to nuclear replication centers.

Our data show a strong correlation between the expression and nuclear localization of the RV2 ORF59 proteins and the replication of viral genomes and production of infectious virus during RRV infection of RPFF cells. This confirms previous studies that RRV infection of rhesus fibroblasts cells is permissive (Desrosiers et al., 1997; DeWire et al., 2003). Our studies demonstrate that Vero cells are similar to RPFF cells in their ability to be infected by and support the replication of RRV. The RRV-infected Vero cell cultures showed high levels of nuclear ORF59-positive cells, replicated viral genomes and infectious virus released into the culture medium. The level of cell-associated viral genomes at 24 and 48 hours was comparable to that seen with RPFFs, but the level did not increase as significantly over time. The amount of cell-free virus in the culture medium of the infected Vero cells mirrored the results seen with infected RPFF cells. Interestingly, the ORF59-positive RRV-infected Vero cells displayed evidence of aggregation and syncytia formation that was not evident with the RRV-infected RPFF cells. Further analysis of this phenomenon is ongoing. Our data do not agree with a previous study, which indicated that Vero cells did not support RRV replication (DeWire et al., 2003). The reason for this discrepancy is not clear, however, we utilized the 17577 isolate of RRV in our study, while the DeWire study utilized the H26-95 isolate [26].

Previous microarray analysis has shown that RRV ORF59 mRNA expression initiates 12-24 hours post infection and the levels increase significantly between 48 and 72 hours. RRV ORF59 mRNA expression during this time course was significantly inhibited by treatment of cells with phosphonoacetic acid (PAA) suggesting that the *orf59 *gene could be classified as an early-late viral gene whose expression is dependent upon viral replication[34]. KSHV *orf59 *gene expression is induced 10-24 hours after TPA induction of latently-infected PEL cells [18,39], and mRNA abundance was maximal 48 hours post-induction and with subsequent decline thereafter [18]. PAA treatment blocked the majority of KSHV ORF59 protein synthesized at 24 hours, indicating that it also belongs to the early-late class of viral proteins [18]. We analyzed the expression of RRV ORF59 mRNA after RRV infection of RPFF cells using real-time quantitative RT-qPCR and compared it to the expression of several other RRV genes. RRV ORF59 mRNA expression was first detected 8 hours post infection, similar to the time frame for KSHV ORF59 mRNA expression after TPA induction of latently infected cells. We found that RRV ORF59 mRNA expression was concomitant with the expression of the mRNA for the ORF50 viral activator, the ORF9 DNA polymerase, and the ORF8 glycoprotein B. The co-expression of these genes at early time points after RRV infection of RPFF cells is consistent with a permissive RRV infection and the onset of viral replication and production of infectious virions.

Using our rabbit RV2 ORF59 antiserum, we detected RV2 infections in keratinocytes within the differentiated epithelium of the skin of an MneRV2-infected pig-tailed macaque. This is the first evidence for an RV2 rhadinovirus infection of a non-lymphoid cell *in vivo*. The anti-RV2 ORF59 antiserum gave low level and intermittent staining of the columnar keratinocytes in the basal layer of the skin epithelium in this animal. Cells in this layer consist of stem cells and transit-amplifying cells, which are continuously dividing. These cells are the source of the suprabasal cells that have migrated into the upper epithelial layers and undergone terminal differentiation [40]. In our study, we observed consistent and strong nuclear ORF59 staining in the nuclei of keratinocytes present in the more differentiated spinous and granular layers of the epithelium. Whereas cells in the upper epithelium of uninfected animals typically lose their nuclei [40], we observed RV2 ORF59-positive nuclei throughout all layers of the infected epithelia. This is reminiscent of papillomavirus-infected epithelia where the infected cells leave the basal layer and remain active in the cell cycle due to the action of the E7 oncoprotein [41]. As the papillomavirus-infected cells differentiate during their migration from the basal layer, the replicative cycle of the virus is activated with high-level expression of transcripts encoding viral replication-associated proteins and late gene products. The viral genomes are replicated and the virions are assembled with the release of the mature virions from nucleated cells present in the uppermost layers of the epithelium. Our results suggest that a similar phenomenon is occurring in the epithelial cells infected with the RV2 rhadinoviruses. As in the case of the RPFF and Vero cells undergoing permissive *in vitro *infection with the RV2 rhadinoviruses, the suprabasal epithelial cells in the skin of the MneRV2 infected macaque were strongly reactive with the anti-RV2 ORF59 antiserum in nuclei. In KSHV infected cells, nuclear localization of ORF59 drives nuclear import of the KSHV DNA polymerase during activation of the replicative cycle of the virus [20]. Our *in vitro *results demonstrate a strong correlation of the expression and nuclear localization of RV2 ORF59 with the replication of the viral genome and production of infectious virions. Our *in vivo *ORF59 staining results demonstrate that suprabasal keratinocytes in differentiated skin epithelium are infected with the RV2 rhadinovirus and express nuclear ORF59 suggesting that they are actively replicating the viral genome and producing infectious virions. Verification of this is ongoing.

Infectious KSHV virions are detected in the saliva of infected individuals, and saliva is thought to be the major route of transmission for *de novo *infections [42,43]. *In vitro *tissue culture models support a role for differentiation of latently infected oral epithelial cells in the activation of KSHV replication and production of infectious virions in saliva [44]. We have also detected RV2 rhadinovirus genomes in macaque saliva suggesting a similar mode of transmission (unpublished results). Our immunohistochemical results indicate that epithelial differentiation is also an activating event to initiate virus replication and production of infectious RV2 virions in latently infected cells. The activation of virus replication detected in our study would likely lead to the production and release of infectious RV2 virions from skin epithelium in addition to virus released into saliva from the oral epithelium. Current studies are ongoing to determine whether skin epithelia plays a role in RV2 transmission, as occurs with papillomavirus infections.

## Conclusion

The ORF59 DNA polymerase processivity factor homologs of the Old World primate RV1 and RV2 rhadinovirus lineages are phylogenetically distinct yet demonstrate similar expression, and localization characteristics that correlate with their use as lineage-specific markers for permissive infection and virus replication. The development of an antiserum specific for the ORF59 homologs of macaque RV2 rhadinoviruses provides an approach to studying the permissivity of RV2 rhadinovirus infection in cell cultures *in vitro *and in the macaque *in vivo*. This will aid in the characterization of virus activation from latency to the replicative state, an important step for understanding the biology and transmission of rhadinoviruses, such as RRV and KSHV.

## Materials and methods

### Tissue

Retroperitoneal fibromatosis (RF) tumor tissue from *M. nemestrina *(MneM78114), naturally infected with RFHVMn and MneRV2, was provided by C.-C. Tsai, Washington National Primate Research Center (WaNPRC). Spleen tissue from Mne442N, an *M. nemestria *naturally infected with RFHVMn and MneRV2, was obtained from R. Shibata, National Institutes of Health, Bethesda, MD. RF tumor tissue from *M. mulatta *(MmuYN91-224), naturally infected with RFHVMm, was kindly provided by the late H. McClure (Yerkes National Primate Research Center). Spleen tissue from an *M. fascicularis *(Mfa95044) naturally infected with RFHVMf and MfaRV2 was obtained through the Tissue Distribution Program at the WaNPRC. Tissue samples from a chimpanzee naturally infected with PtrRV1 and PtrRV2 were a gift from S. Shapiro at M.D. Anderson. Formalin-fixed paraffin-embedded tissue blocks were obtained from a female *M. nemestrina *(MneF94292) (WaNPRC) that was naturally infected with simian retrovirus-2 and MneRV2.

### Mammalian Cell Culture

Rhesus primary fetal fibroblasts (RPFF) were kindly provided by M. Axthelm, Oregon National Primate Research Center (ONPRC) and grown in DMEM complete (Invitrogen) at 37°C, 5% CO_2_. African green monkey kidney epithelial cells (Vero) were obtained from M.E. Thouless, University of Washington and grown in DMEM complete at 37°C, 5% CO_2 _.

### Rhadinovirus isolates

An isolate of the rhesus macaque RV2 rhadinovirus, RRV strain 17577, was kindly provided by M. Axthelm and S. Wong (ONPRC). The MneRV2 isolate J97167 obtained from an *M. nemestrina *at the WaNPRC was described previously [30]. Virus stocks were prepared by infecting cultures of RPFF cells with either MneRV2 or RRV. Viral particles were harvested from culture supernatant by high speed centrifugation. or by concentration on a 50% Opti-Prep (Iodixanol) cushion [45]. Virus stock was passed through a 0.45 μm filter prior to use.

### OFR59 cloning and sequence analysis

The protein sequences of the ORF58, ORF59 and ORF60 genes from KSHV and RRV (NCBI) were aligned using ClustalW. The consensus-degenerate hybrid oligonucleotide primer (CODEHOP) technique [46-48] was used to design CODEHOP PCR primers from conserved amino acid motifs within these genes to enable the amplification and sequence analysis of this region within other Old World primate RV1 and RV2 rhadinoviruses. Primers NFFEa (ORF60) and PQFVb (ORF59) (see Table 1) were designed based on the homology between KSHV and RRV and used to amplify a portion of the *orf59-60 *intergenic region of RFHVMn from spleen DNA from Mne442N. Amplification reactions were performed on an iCycler (BioRad) with Platinum TAQ (Invitrogen) using the supplied buffer, 2 mM MgCl_2 _and a 55-70°C temperature gradient. From the RFHVMn *orf59 *sequence obtained with these primers, a specific primer, YGVRb, was identified (Table 1). A CODEHOP PCR primer, WCFIb (Table 1), designed from conserved sequence motifs identified in the *orf58 *homologs of KSHV and RRV, was used with the YGVRb primer to amplify the remainder of the *orf59 *allowing the determination of the complete RFHVMn ORF59 sequence. A similar approach was used to amplify the complete ORF59 genes from RFHVMm, RFHVMf, PtrRV1, MneRV2, MfaRV2, PtrRV2 and human EBV (BMRF1) using the primers shown in Table 1.

### Sequence alignment and phylogenetic analysis

Protein sequences were aligned using ClustalW and analyzed using the protein maximum-likelihood program from the Phylip package, version 3.62 (University of Washington, Seattle). Phylogenetic tree output was produced using TreeView (R.D.M. Page).

### RT-qPCR assay for RRV mRNA expression

RPFF cells were plated at 2 × 10^5 ^in a 6-well plate and grown overnight at 37°C, 5% CO_2_. The cells were incubated with purified RRV (MOI of ~0.01) for 2 hours, washed several times and then further incubated at 37°C for various times. The cells were harvested with trypsin and pelleted by centrifugation. RNA was isolated using an RNeasy kit (Qiagen) following the manufacturers instructions, treated with DNAase to remove contaminating viral genomic DNA and converted to cDNA (250 ng of DNAase-treated RNA, 50 pmoles of random decamer primers (IDT), 0.5 mM dNTPs, M-MLV buffer (Promega) and 1 unit M-MLV reverse transcriptase - 50 minutes at 42°C followed by inactivation at 70°C for 15 minutes). SYBR green-based qPCR assays were developed to quantitate RRV mRNA levels for ORF8 (glycoprotein B), ORF9 (DNA Polymerase), ORF50 (viral transactivator), ORF73 (latency-associated nuclear antigen) and ORF59 (DNA polymerase processivity factor). Amplification primers for each gene were derived from the published RRV17577 genome sequence (NC_003401) (Table 4) [14]. PCR amplification of cDNA was performed with IQ SYBR Supermix (BioRad) with a 95°C denaturation step for 30 seconds, a 62°C annealing step for 30 seconds and elongation at 72°C for 30 seconds for 50 cycles. The presence of contaminating viral DNA in the DNAase-treated RNA was controlled for by eliminating reverse transcriptase in the cDNA conversion. All of the assays were found to be > 95% efficient based on standard curves using 4-fold dilutions of DNA purified from RRV infected RPFF cells. Viral mRNA expression was normalized to the cellular ribosomal phosphoprotein (RPO) mRNA levels detected by RT-qPCR (see Table 4 for primers) using the delta cycle threshold (ΔCT) method.

**Table 4:** Oligonucleotide primers for RT-qPCR amplification of RRV (17577) genes

Gene	Orientation	Name	Sequence (5'-3')^1^

ORF8 (Glycoprotein B)	Forward	IQTTa	CAGGGCGATACAGACGAC

	Reverse	ATIKb	TCTTGATGGTGGCGTTGA


ORF9 (DNA Polymerase)	Forward	HGFTa	GGATCACGGGTTGACCAC

	Reverse	TREGb	GCCTTCCCTGGTCGTGT

ORF50 (Replication and Transcription Activator)	Forward	ATCNa	CCGCCACCTGTAACGTC


	Reverse	SFPRb	CCTTCATTTCCGCGTAG


ORF59 (DNA Polymerase Processivity Factor)	Forward	HKSVa	GTGCACAAGAGCGTCGTG

	Reverse	CKIPb	ATCCCGGAATCTTACATG


ORF73 (Latency-associated nuclear antigen)	Forward	RGGTa	CGCGGCGGCACTAGA

	Reverse	MATLb	CCTGATGGCGACCCTT


Cellular Ribosomal Phosphoprotein (RPO)	Forward	RP01a	AGCAGGTGTTCGACAATGGCA

	Reverse	RP01b	ACTCTTCCTTGGCTTCAACC

^1 ^Primer sequences derived from RRV (17577) accession [NC_003401]

### Bacterial expression of ORF59 proteins

pGEX-2T and pQE30 vectors were constructed to express ORF59 homologs from different RV1 and RV2 rhadinoviruses fused to either GST or 6X-His, respectively. A sense primer containing a BamHI site followed by the 5' coding sequence downstream of the initiation codon ATG was designed for each ORF 59 homolog except for PtrRV1 and PtrRV2 for which the primers contained BglII sites. Anti-sense primers were derived from the 3' end of the individual ORF59 genes, and contained a stop codon and a HindIII site with the exception of the PtrRV1 primer which had a BglII site. The primers were used to amplify the *orf59 *genes from the DNA templates shown in Table 1 with a temperature gradient as described above. The PCR products were digested with the appropriate restriction enzymes and inserted into BamHI digested pGEX-2T(PtrRV1) or BamHI and HindIII digested pGEX-2T to produce GST-ORF59 fusion expression clones. The same PCR fragments were inserted into digested pQE30 vector to produce 6X-His ORF59 fusion expression clones. The resulting plasmids were transfected into Rosetta 2 cells (pGEX-2T) or M15 cells (pQE30). Bacteria were grown to an OD_600 _of 0.7-1.0, induced with 0.2 mM IPTG and incubated for 5 hours. GST-fusion proteins were obtained by sonication of the cell pellets, adsorption onto s-hexylglutathione-agarose (Sigma) and elution with 20 mM glutathione. 6XHis fusion proteins were obtained by lysis of the bacterial cell pellets in 6 M guanidine, adsorption onto Ni-NTA-agarose (Qiagen), washing with 1 M urea pH 6.3 and elution in 1 M urea pH 4.5.

### Preparation of rabbit polyclonal anti-RV2 ORF59 antisera

The coding sequences for amino acids 300-388 of RRV(17577) and MneRV2(442N, this study) ORF59 were each inserted into the pQE30 expression vector to produce N-terminal 6XHis-tagged ORF59 fusion polypeptides. Recombinant proteins were prepared in M15 bacteria, as described above. The two ORF59 polypeptides were purified on Ni-NTA-agarose and reverse-phase HPLC, combined as a single immunogen, and injected into two rabbits, 424 and 425.

### Western analysis

The ORF59 recombinant proteins were analyzed on Invitrogen 4-12% Bis-Tris SDS-PAGE gels in MES buffer and transferred to PVDF membranes. The membranes were blocked with tris-buffered saline with 0.1% Tween-20 and 5% non-fat milk prior to incubation with either a 1:1000 dilution of macaque plasma, a 1:10,000 dilution of 425 anti-RV2 ORF59 rabbit polyclonal serum, a 1:5,000 dilution of mouse monoclonal anti-GST (Sigma), or a 1:10,000 dilution of mouse monoclonal anti-RGS 6X His (Qiagen). The membranes were then incubated with a 1:10,000 dilution of anti-human IgG-Dylight 700 for macaque serum, anti-rabbit IgG-DyLight 800 or anti-mouse IgG-Dylight 800 (Thermo Scientific). The immunoreactive proteins were visualized on an Odyssey Infrared Imager (Li-Cor). The Pageruler Plus prestained protein ladder (Fermentas) was used for molecular weight estimation.

### ORF59 immunofluorescence assays

Approximately 1.5-2.0 × 10^4 ^cells were plated onto 17 mm spots in 60 mm Petri dishes in 100 μL of complete DMEM and were incubated overnight at 37°C. The cells were infected at low MOI (100 μL of purified RRV or MneRV2 at a dilution of 1:10) for 2 hours at 37°C. Virus was removed and 150 μL fresh media was added to each spot. Cells were incubated for various times prior to fixation in 8% paraformaldehyde. Cells were stained with a 1:1000 dilution of HHV8-ORF59 mouse monoclonal (ABI) or a 1:650 dilution of polyclonal rabbit 425 anti-RV2 ORF59 antiserum. For visualization of ORF59, cells were stained with a 1:500 dilution of goat anti-mouse IgG-Alexa 594, or goat anti-rabbit IgG-Alexa 488 (Invitrogen). To identify nuclei, cells were stained with a 1:500 dilution Topro-3 (Invitrogen) before the last fixation. Images were viewed using a Zeiss LSM 5 Pascal confocal microscope.

### RRV replication and production of infectious virions

RPFF or Vero cells were plated at 2 × 10^5 ^in a 6-well plate and grown overnight at 37°C, 5% CO_2_. The cells were incubated with purified RRV (MOI of ~0.01) for 2 hours, washed several times and then further incubated at 37°C. At 1, 2, 3, 5 and 8 days post infection, the culture medium was collected and the cells were harvested by trypsinization and low-speed sedimentation. DNA was extracted from each cell pellet and from the combined culture medium obtained from each well during the total incubation period. The levels of RRV DNA in the cultured cells and medium were determined by real-time qPCR as described [30]. To demonstrate the production of infectious virus in the cell cultures, culture medium was removed from the RRV-infected RPFF and Vero cell cultures 5 days post-infection and incubated with uninfected RPFF cell cultures for 2 hours. The cell cultures were washed several times and further incubated. Five days post infection, the level of RRV DNA in the culture medium was determined by qPCR.

### Immunohistochemistry

Tissue slides were deparaffinized and heated in Target Retrieval, pH 9 (DAKO) for 25 min followed by 20 min at room temperature. This was followed by blocking steps (0.3% H_2_O_2 _for 10 min, 0.15 M glycine/PBS for 15 min, 1% BSA and 10% normal horse serum in TBST (Tris-buffered saline with 0.2% Tween-20) for 30 min). The slides were then incubated with the 425 rabbit anti-RV2 ORF59 antiserum (1:300 in TBST/BSA/NHS) for 2 h at room temperature. Bound antibody was visualized with the Envision kit from DAKO. All incubation steps were interspersed with washing-steps using TBST (3 × 5 min).

## Abbreviations

RV1: rhadinovirus 1 lineage; RV2: rhadinovirus 2 lineage; KSHV: Kaposi's sarcoma-associated herpesvirus (human herpesvirus 8); PtRV1: *Pan troglodyte *rhadinovirus 1; PtRV2: *Pan troglodyte *rhadinovirus 2; RFHVMn, Mm, Mf: Retroperitoneal fibromatosis-herpesvirus from *Macaca nemestrina, Macaca mulatta, Macaca fascicularis*; RRV: Rhesus rhadinovirus; MneRV2: *Macaca nemestrina *rhadinovirus 2; MfaRV2: *Macaca fascicularis *rhadinovirus 2; RPFF: rhesus primary fetal fibroblasts

## Competing interests

The authors declare that they have no competing interests.

## Authors' contributions

Design and conception of the study (AGB, TMR); development of the methods and amplification of the ORF59 homologs (AGB, AMB); Development of the RT-qPCR assays and quantitative analysis (AGB, CAG); Virus infection studies (AGB, CAG, KLB); Sequence analysis, alignment and phylogeny (AGB, TMR); Immunofluorescence studies (CAG, KLB, LKD); Immunohistochemical analysis (HBO); Antiserum development (AGB, AMB, TMR); Protein purification and Western analysis (AGB; AMB; CAG); Manuscript preparation (AGB, CAG, LKD, HBO, KLB TMR). All authors read and approved the final manuscript.
